# Young adult preferences for digital health interventions to support adherence to inhaled corticosteroids in asthma: a qualitative study

**DOI:** 10.1080/21642850.2022.2085709

**Published:** 2022-06-16

**Authors:** Jane Murphy, Gerard J. Molloy, Lisa Hynes, Jenny McSharry

**Affiliations:** a Medication Adherence Across the Lifespan Research Group, School of Psychology, National University of Ireland, Galway, Ireland; b Croí, The West of Ireland Cardiac Foundation, Galway, Ireland; c Health Behaviour Change Research Group, School of Psychology, National University of Ireland, Galway, Ireland

**Keywords:** Digital health interventions, medication adherence, self-management, asthma, young adults

## Abstract

**Objective:** Adherence to inhaled corticosteroids (ICS) among young adults living with asthma is low and in need of appropriate intervention. Digital health interventions (DHIs) have demonstrated potential to improve ICS adherence; however, young adult preferences for these DHIs and how their use could support adherence in this population remain understudied. Therefore, this study aimed to explore young adult preferences for ICS adherence supports and potential DHI features to deliver these supports, in order to improve adherence behaviour throughout this critical developmental stage of the lifespan.

**Methods:** Qualitative, semi-structured interviews were conducted with 13 young adults living with asthma. Analysis followed an inductive, reflexive thematic approach.

**Results:** Participant’s age ranged from 18 to 30 years (*M *= 24.7; 8 female). Three themes were developed from the analysis: ‘Enabling young adults to find their ‘own way of knowing', ‘Support for making a habit of adherence’, and ‘Providing accessible information’ which included the sub-themes: ‘Education on asthma self-management and medication’, ‘Self-monitoring information’ and ‘Personal feedback on outcomes of adherence’. Suggested features to deliver these supports included a medication and prescription refill reminder, adherence charts, symptom and trigger monitoring, rewards for adherence, visual representations of lungs demonstrating the impact of adherence and lung function monitoring.

**Conclusion:** DHIs may offer an appropriate solution to improve suboptimal adherence to ICS in young adults. However, it is crucial that young adult preferences for adherence supports and features are integrated into these interventions in order to optimise engagement and support adherence behaviour in this population.

## Introduction

Asthma represents the most prevalent chronic respiratory disease affecting 339 million people worldwide (Asthma Society of Ireland (ASI), 2019; Vos et al., 2017). Over 60% of those living with the condition have uncontrolled symptoms (Centers for Disease Control and Prevention (CDC), 2016; ASI, 2018), resulting in reduced quality of life, increased productivity loss, healthcare use and related mortality (Gruffydd-Jones et al., 2019; Gullach et al., 2015; Nunes, Pereira, & Morais-Almeida, 2017; Stridsman, Axelsson, Warm, & Backman, 2020). Asthma control can be maintained through effective self-management (Eakin & Rand, 2012; Global Initiative for Asthma (GINA), 2021; British Thoracic Society, 2019) with adherence to appropriate treatment being the cornerstone behaviour. The Global Initiative for Asthma (GINA) recommend the use of Inhaled Corticosteroids (ICS), which are deemed the most effective controller medication for asthma (Barnes, 2010; Kearns, Maijers, Harper, Beasley, & Weatherall, 2020; Liang & Chao, 2017; O'Byrne et al., 2019), to optimally manage the condition (GINA, 2021). The clinical benefits of adherence to ICS are well established including increased asthma control and lung function, and reduced exacerbations and healthcare use (Bårnes & Ulrik, 2015; George & Bender, 2019; Mäkelä, Backer, Hedegaard, & Larsson, 2013; Williams et al., 2011). Despite these benefits, suboptimal adherence remains a significant contributor to uncontrolled asthma, particularly in earlier stages of the lifespan (Kaplan & Price, 2020; Klok, Kaptein, & Brand, 2015).

Increasing evidence has demonstrated that a substantial discrepancy exists between GINA-based guidelines and actual management of asthma in young adults in many healthcare contexts (Dahlén et al., 2021; Ödling et al., 2020). A recent meta-analysis revealed that only 28% of young adults adhere to their ICS as prescribed (Murphy et al., 2020). Adherence to ICS may be challenging for this population as they move from asthma management with parental/caregiver support to independent self-management (Orrell-Valente, Jarlsberg, Hill, & Cabana, 2008). Alongside this transition, young adults typically experience a distinct stage of psychological development that has been defined as Emerging Adulthood (Arnett, 2000, 2014). Emerging Adulthood occurs between adolescence and adulthood spanning age 18–29 years, during which young adults often become more autonomous, explore possibilities in education, work, relationships and residence (Arnett, 2000, 2014) and participate more in health risk behaviours (Claxton & van Dulmen, 2013; Krieger, Young, Anthenien, & Neighbors, 2018; Li, Simons-Morton, Gee, & Hingson, 2016; National Institute on Drug Abuse, 2017). The duration and relevance of this developmental phase is likely increasing across many cultural contexts as young adults are typically spending longer in education to pursue occupations with increasing credential requirements (Schwartz & Petrova, 2019), leading to a rising average age of relevant milestones such as home ownership, marriage and parenthood (Eurofound, 2019; Fry, 2016; Vespa, 2017). Living with asthma adds to this already challenging, dynamic period, by requiring young adults to navigate between the typical developmental demands of transitioning to young adulthood and the self-management tasks required to maintain asthma control (Withers & Green, 2019). Recent evidence has demonstrated that attempting to balance these challenges impacts adherence to ICS specifically (Zaeh et al., 2021). Therefore, developmentally appropriate solutions may be needed to support optimal self-management, especially adherence to ICS, throughout this stage of the lifespan.

It is recognised that young adults require asthma self-management and adherence supports beyond what is typically provided in many healthcare contexts internationally, and which they can regularly access and use (Roberts et al., 2020). Digital technologies may offer a suitable intervention medium to deliver these accessible supports given their widespread use across young adult populations (Taylor & Silver, 2019). For example, young adults are the most likely cohort to own a smartphone and use the internet in comparison to all other age groups in both high and low-middle income countries (Taylor & Silver, 2019). Furthermore, a recent review identified specific predictors of non-adherence to ICS in young adults that may need to be addressed with such accessible supports, including medication concerns, and lack of a medication routine, knowledge, and a perceived need for ICS (Murphy et al., 2020). Accumulating evidence indicates that digital health interventions (DHIs) for asthma may offer effective solutions to several of these predictors in adolescents (Alquran et al., 2018), children and adults (Jeminiwa et al., 2019; Miller, Schüz, Walters, & Walters, 2017; Unni, Gabriel, & Ariely, 2018).

Despite the potential for asthma DHIs to be particularly appropriate for a young adult population, little is known about young adult preferences for these interventions and how their use could support adherence to ICS in this specific population. This may be due to a lack of young adult involvement throughout the DHI development process. Across 3 recent reviews of asthma DHIs for all age cohorts (Jeminiwa et al., 2019; Miller et al., 2017; Unni et al., 2018) only one intervention out of a collective total of 66 was developed with young adults (MacDonell, Naar, Gibson-Scipio, Lam, & Secord, 2016). Additionally, more recent developments in technology capabilities e.g. enriched interactive video content, beyond the earlier types of digital interventions included in these reviews, will likely impact young adult preferences for asthma DHIs. Increased young adult involvement in the development process is essential to meet the unique, understudied needs of this population (Withers & Green, 2019).

To address the lack of attention given to young adults within this context, this study aims to explore young adult preferences for ICS adherence supports and potential DHI features to deliver these supports. In line with existing guidance for DHI development (Yardley, Morrison, Bradbury, & Muller, 2015), we selected a qualitative approach as it is considered most appropriate for exploring under-researched topics within the target user population and to understand and accommodate these perspectives. Identifying young adult preferences for DHIs would be highly conducive to informing the development of appropriate solutions to support adherence to ICS throughout this important developmental stage of the lifespan.

## Method

### Design

Individual, semi-structured qualitative interviews were conducted and analysed using a reflexive thematic analysis approach (Braun & Clarke, 2006, 2019). Ethical approval was granted by the National University of Ireland, Galway, Research Ethics Committee (Ref: 18-Dec-16). This study is reported in accordance with the Consolidated criteria for Reporting Qualitative research (COREQ) checklist (Tong, Sainsbury, & Craig, 2007). A completed COREQ checklist is included in Appendix 1.

### Sampling and recruitment

Eligible young adults were aged 18–30 years old with a self-reported diagnosis of asthma and current prescription for ICS. We defined young adults as 18–30 years for the following reasons: (1) to encompass the Emerging Adulthood age range of 18–29 years (Arnett, 2000, 2014), (2) based on varying definitions of young adults across the self-management and healthcare transition literature (Pai & Ostendorf, 2011; Zhou, Roberts, Dhaliwal, & Della, 2016), and (3) there is precedent for using this definition of young adults in a similar context (Hilderson et al., 2013). Young adults were invited to participate through social media, sports clubs, a university student mailer and study advertisements in gyms, libraries and manufacturing companies. Interested potential participants contacted the first author directly and were asked to provide demographic information including age, gender and education level. Purposive sampling was then used in an attempt to obtain variation along these domains.

Sample size in qualitative studies has typically been discussed with reference to data saturation, defined as the point in which no new information emerges from the data (Given, 2015; Lincoln & Guba, 1985). However, recent literature has demonstrated that this concept is not consistent with the values of reflexive thematic analysis, in which meaning is generated through the researcher’s interpretation of and not simply extracted from the data (Braun & Clarke, 2019). As a result, reflexive thematic analysis does not reach a fixed endpoint and new meanings are always possible. Accordingly, a recommended alternative known as information power was applied to this study (Braun & Clarke, 2019; Malterud, Siersma, & Guassora, 2016). Information power proposes that the more relevant information the sample holds, fewer participants are needed. It recommends that a lower and upper sample size is estimated by the researcher(s) based on the study aim, sample specificity, use of theory, quality of dialogue (based on interviewer’s topic expertise and experience) and analysis strategy (Malterud et al., 2016).

Based on these considerations, the research team estimated that 12–16 interviews may generate sufficient information power for this study. It is recommended that the researchers’ appraisal of information power is repeated throughout data collection, supported by preliminary analysis (Malterud et al., 2016). This enables the researcher to determine when the sample holds sufficient information to address the study aim, and thus decide on the final sample size. In this study, the researcher appraised the information power of the sample after transcribing and preliminary reviewing the data at four timepoints: after 3, 7, 10, and 13 interviews were conducted. After data were collected through 13 interviews, the research team felt the dataset was highly relevant and sufficient to present young adult preferences for adherence supports and DHI features to deliver these supports.

### Procedure

Interviews were held in person on university campus (*n* = 4) or by telephone (*n* = 9) depending on participant preference, from June to August 2019. Interviews were conducted and informed consent was obtained by the first author, a female PhD candidate in Health Psychology trained in qualitative research methods. Participant’s provided information on demographics and asthma characteristics (please see Appendix 3 for the information collected and Table 1 for the results). Asthma control was measured using the 5-item Asthma Control Test (ACT; Nathan et al., 2004; Schatz et al., 2006). ACT scores range from 5 to 25, with higher scores reflecting greater asthma control. An ACT score ≤19 indicates poorly controlled asthma, while a score >19 indicates controlled asthma (Nathan et al., 2004). Adherence to ICS was measured using the 5-item Medication Adherence Report Scale (MARS-5; Horne & Hankins, 2004). MARS scores range from 5 to 25, with higher scores indicating greater adherence. A score of ≥23 indicates adherence (Norberg, Sjölander, Glader, & Gustafsson, 2022).

**Table 1.** Participant characteristics.

**Demographics**	
Age in years, Mean (SD), Range	24.7 (3.9), 18–30
Gender, *N* (%)	
Female	8 (61.5)
Male	5 (38.5)
Ethnicity, *N* (%)	
White: Irish	13 (100)
Education, *N* (%)	
Second level	5 (38.5)
Tertiary/Higher level	8 (61.5)
Medical card holder*: *N* (%)	
Yes	4 (30.8)
No	9 (69.2)
**Asthma characteristics**	
Asthma control, *N* (%)	
Poorly controlled asthma: ACT ≤19*	4 (30.8)
Controlled asthma: ACT >19	9 (69.2)
Total ACT, M (SD), Range	19.7 (3.9), 11–25
Visited ED in last year:	11 (84.6)
Yes	2 (15.4)
No	11 (84.6)
Admitted to hospital in last year, *N* (%)	
No	13 (100)
Prescribed oral steroids in last year, *N* (%)	
Yes	5 (38.5)
No	8 (61.5)
Adherence	
Total MARS*, M (SD), Range	16.5 (5.9), 8–24

Notes: *In the Republic of Ireland, a medical card holder has access to free General Practice services, community health services, dental services, prescription medicines and hospital care. Qualification for a medical card is means-tested. *ACT scores range from 5 to 25, with higher scores reflecting greater asthma control. An ACT score ≤19 indicates poorly controlled asthma, a score >19 indicates controlled asthma. *MARS scores range from 5 to 25, with higher scores indicating greater adherence. A score of ≥23 indicates adherence.

The first author began each interview by providing an overview of the research and reasons for conducting the study. Predictors of non-adherence/adherence to ICS identified in a recent review conducted by the first author (Murphy et al., 2020) were used to devise a semi-structured interview guide (Appendix 2) to elicit discussion of potential adherence supports, digital technology use and preferences. This was used to flexibly guide the interviews with the overall direction led by the participant. Interviews began with general questions about asthma and technology use, followed by more specific questions and prompts for clarification and examples. Only the participant and first author were present during each interview. No relationship existed between the first author and the participants. The interviews were audio-recorded, transcribed verbatim and checked for accuracy by the first author, but were not returned to participants for comment or correction. Transcripts were anonymised and uploaded to NVivo 12 software for data organisation, management, and analysis.

### Data analysis

The analysis was driven from the data itself, following an inductive, reflexive thematic analysis approach (Braun & Clarke, 2006, 2019). No theory, structure or framework was used in the analysis due to the exploratory nature of the study. Analysis was undertaken from a subtle realist epistemology perspective. This perspective recognises the subjective nature of knowledge while retaining a belief in the existence of an underlying reality that we seek to represent through research (Mays & Pope, 2000).

The first author engaged in data immersion by reading, re-reading and coding all transcripts. Initial codes were collated into potential themes relevant to the entire dataset by the first author. Two authors (JMS and LH) checked a randomly selected transcript to ensure data were accurately represented by the proposed themes, followed by research team discussions to further refine these themes. Analysis was iterative with codes, themes and subthemes refined throughout. All refinements were discussed with the research team to ensure credibility of the analysis. Participants were not involved in the analysis or in providing feedback on the accuracy of the findings. Reflective analytic memos were made to document the development of this process.

## Results

Seventeen young adults with asthma expressed an interest in participating. No response was received from two participants. Of the remaining 15, 13 (8 female) were selected for interview based on variation in gender and education. Interviews lasted between 10 and 34 minutes (*M* = 20.36). Participants ranged in age from 18 to 30 years (*M *= 24.7). Table 1 represents participant characteristics and provides an indication of the heterogeneity of the sample.

Three main themes were developed from the analysis: (1) Enabling young adults to find their ‘own way of knowing’, (2) Support for making a habit of adherence, and (3) Providing accessible information. The third theme included the following three subthemes: (3.1) Education on asthma self-management and medication, (3.2) Self-monitoring information, and (3.3) Personal feedback on outcomes of adherence. Table 2 illustrates themes and sub-themes, while Table 3 presents themes and the corresponding features that were suggested by participants to deliver adherence supports.

1. Enabling young adults to find their ‘own way of knowing’

**Table 2.** Themes and sub-themes.

Theme	Sub-theme
(1) Enable young adults to find their ‘own way of knowing’	
(2) Support making a habit of adherence	
(3) Provide accessible information that is ‘100% missing’:	(3.1) Education on asthma self-management and medication
	(3.2) Self-monitoring information
	(3.3) Personal feedback on outcomes of adherence

**Table 3.** Themes and suggested digital health technology features.

Theme	Suggested feature(s) to deliver this support
(1) Enable young adults to find their ‘own way of knowing’	Prescription refill reminder
(2) Support making a habit of adherence	Medication reminder with succinct information on the importance of adherence Prescription refill reminder
(3) Provide accessible information that is ‘100% missing’:	
(3.1) Education on asthma self-management and medication	Images/videos from HCPs with concise text on asthma and ICS benefits
(3.2) Self-monitoring information	Adherence chart Symptom and trigger monitoring Rewards for adherence
(3.3) Personal feedback on outcomes of adherence	Visuals of lungs before and after ICS adherence Lung function monitoring

This theme centres on participants’ desire for support in a DHI to increase their autonomy to take responsibility for adherence and self-management. Participants expressed how the high prevalence of asthma contributes to a common perception of it being a trivial disease that does not have a significant impact on quality of life, and that such perceptions prevent young adults from seeking self-management support: ‘everyone just seems to be diagnosed with it. So, it means we brush it off as just not that big a deal when it actually would be when we don't take care of it*’* (Participant (P.) 12; female, age 19, second level education). They valued the prospect of having a DHI as a self-management support that they could independently access and use.

There was an appreciation for the potential to increase autonomy from healthcare providers (HCPs) by having access to personal information on adherence behaviour and asthma outcomes, and reliable information on the condition. This would have an additional benefit of being more convenient and cost-effective than visiting the doctor ‘because, that costs money and stuff’ (P. 7; female, age 30, tertiary/higher level education):

I’d certainly want to check in on how I'm doing every so often, rather than going to the doctor or the pharmacy. Like if I had my own way of knowing, where I'm at with it, obviously if I'm struggling I'd go to them, but … just to reassure yourself that you're on top of it. (P. 12; female, age 19, second level education)

A DHI may also increase young adults’ autonomy from their parents by enabling them to take responsibility for medication renewal. A prescription refill reminder was mentioned as an appropriate support to facilitate the transition of this responsibility, while simultaneously improving timely medication renewal and therefore adherence: ‘I forget to say it to mam and she wouldn't notice. She wouldn’t be looking. So I'd run out and then could be out of an inhaler for 2 days or so’ (P. 11; male, age 18, second level education). Although some participants already had taken this responsibility, they often forgot when a refill was due and so would also value this support within a DHI: ‘it's easy to forget … sometimes I get to the end and not realize until I feel wheezy, two days later. So a reminder to go to the pharmacy, would be helpful.’ (P. 1; female, age 28, tertiary/higher level education).

Participants would appreciate the ability to choose when to use certain features and receive notifications based on their preferences and needs at different times: ‘I'd like if they were there, but that they were optional … So it could be, away in the menu, and I wouldn't have to go into it every day.’ (P. 1; female, age 28, tertiary/higher level education). For example, when participants suggested having an asthma ‘check-in’ feature, some indicated that they would want to use this feature regularly ‘like a monthly check in’ (P. 12; female, age 19, second level education), while others may only want to use this feature seasonally ‘when there is allergens in the air, like pollen and that’ (P. 2; female, age 23, second level education). Options for accessing more or less information would also be important, as users would be interested in accessing different amounts of information at different times: ‘maybe a click for more or something … because, someday I could be rushing and want it over and done with. But then another day I could be more willing to have a read’ (P. 12; female, age 19, second level education).

While an app was described as the technology most often used, and so may be the most suitable to support adherence for these participants: ‘Definitely an app, would be more efficient. It's more so what I'd use day to day’ (P. 8; male, age 26, tertiary/higher level education), some participants may not be interested in using any DHI to improve suboptimal adherence, due to a lack of perceived asthma severity. They felt that if their asthma were more severe, its severity alone would be enough motivation to increase adherence without any additional support, ‘I think my asthma is, not that bad. Maybe if it was worse, I would. But then again, maybe if it was worse, I'd be taking the preventer all the time.’ (P. 6; female, age 26, tertiary/higher level education).

(2) Support for making a habit of adherence

Participants discussed how a DHI could facilitate integrating adherence into their daily routine. They related previous positive experiences of using a reminder feature for taking other medication to their ICS and felt this feature may be useful in this context as their only or main barrier to ICS adherence was forgetfulness:

with other alarms I set, it does remind me to take the stuff so … I don't see why an asthma one wouldn't do the same … it's only a case of reminding me like I don't have any issue with taking my preventer, it would just be a case of trying to remember to get into a routine*.* (P. 12; female, age 19, second level education)

They felt that identifying a convenient time or behaviour in their daily routine at which to set the reminder would optimise the likelihood of them taking their ICS at that time by ‘linking it to something you’re doing everyday anyway’ (P. 7; female, age 30, tertiary/higher level education) making the behaviour ‘easier and more consistent’ (P. 3; male, age 26, tertiary/higher level education):

‘like a reminder, around your normal waking and going to bed time, to remind you to take it even if you don't need it.’ (P. 3; male, age 26, tertiary/higher level education)

Moreover, a daily reminder was perceived as an opportunity to deliver ‘small bites of information’ (P. 2; female, age 23, second level education) about why adherence is important. The addition of such information was suggested as a solution to prevent ignoring the reminder, particularly when participants feel like ‘I can breathe right now. I don’t need it*.’* (P. 2; female, age 23, second level education):

something to do with why you're taking it as well as just prompting me to take it. That might help because I would just end up dismissing an alarm. I think it would be good to bring my mind to it. But it might need something more than that as well? (P. 5; male, age 28, tertiary/higher level education)

Participants also perceived worth in inputting and integrating their personal information on their first use of the intervention to receive subsequent ‘smart’ reminders/notifications that would be responsive to their triggers and changes to their daily routine:

put in their demographics, so their ages. And if they're in the age of drinking or going out, on Friday or Saturday evening, a push notification could say, are you heading out tonight? Take your preventer and bring your inhaler with ya. (P. 4; male, age 25, tertiary/higher level education)

Already having a habit of taking ICS was also as a reason why few participants may not feel the need to use a DHI, particularly if they perceive no other barrier to their adherence, ‘* … *for as long as I remember, I've had the routine of brush your teeth and take your inhaler. So, I don't think I'd need something*.’* (P. 1; female, age 28, tertiary/higher level education).

(3) Providing accessible information

The final theme represents participants interest in having an accessible, reliable source of information on asthma and ICS medication. They felt this source of information was missing and that having it would motivate adherence: ‘if I had easy access to that information, the ins and outs of how it works on an app on my phone, I’d be more likely to use this medication, as directed*.*’ (P. 3; male, age 26, tertiary/higher level education).

Participants’ interest pertained to accessing information on three levels, which are described under the following subthemes.

(3.1) Education on asthma self-management and medication

Participants described an interest in general asthma and ICS-specific information such as why daily ICS use is necessary, the correct inhaler technique and relevant potential side effects: ‘if there was a video of your lungs and you could see the medication going in. How it going in everyday helps to keep your airways open.’ (P. 1; female, age 28, tertiary/higher level education).

Participants emphasised that information should come from a reliable source, to increase their engagement with it: ‘I'd prefer if it was a healthcare person, because I'd take it a bit more seriously’ (P. 12; female, age 19, second level education). They would like information to be succinct and easy-to-understand. If information was ‘wordy or technical.. it’d just sound like, school’ (P. 7; female, age 30, tertiary/higher level education) and lead to disengagement. Using a combination of short text and visuals to present this information may accommodate different learning styles: ‘I use diagrams when I'm teaching myself stuff … they would help me’ (P. 12; female, age 19, second level education).

(3.2) Self-monitoring information

Participants want access to their personal adherence and self-management behaviour to identify both controlled and uncontrolled periods of asthma for any necessary intervention: ‘It’d be handy to have something that would tell me, no, you're not doing great at controlling it or yeah, you're actually fine.’ (P. 12; female, age 19, second level education).

Visualising their adherence data would help participants to identify and understand patterns in their behaviour, and subsequently increase motivation to improve adherence:

I find it, motivating when I see my own progress, whether it's going up or down. It’d help me … change or be more conscious of my behaviour if I could see okay, hang on a second, just dipped entirely down here. (P. 5; female, age 28, tertiary/higher level education)

Incorporating this information into a rewards feature was suggested to increase motivation to perform and maintain the behaviour, particularly ‘if someone had more trouble with motivation’ (P. 5; female, age 28, tertiary/higher level education)*:*

I'm such a person for getting a streak on things like if I've gotten something … six days in a row, I'm not gonna want to break that. So like if you took your inhaler every day, you'd get like five in a row.. like collect stars or something … People do like those kind of reward things that they they're on a streak, they haven't missed a day. If you could do that, that would be quite cool. (P. 7; female, age 30, tertiary/higher level education)

Participants would also value having access to this information to learn about their asthma such as triggers and patterns of symptoms: ‘I get wheezy after exercise. And I didn't realize for a while. So if you had a symptom tracker, you'd put those patterns together more easily.*’* (P. 1; female, age 28, tertiary/higher level education).

(3.3) Personal feedback on outcomes of adherence

Participants want information on their asthma outcomes as a result of their adherence behaviour, to understand the effectiveness of the medication. They want feedback on how ICS is benefitting them, which they may not get from the medication alone: ‘You’d be wondering what is it actually doing. If you weren't seeing results or anything. You'd want to know more about it.’ (P. 13; male, age 19, second level education).

Visual representations of lungs before and after increased adherence to ICS would enable users to relate these illustrated benefits to ‘what’s happening inside you’ (P. 7; female, age 30, tertiary/higher level education):

‘show them a pair of lungs and you can see that their airways were quite tight. But if they were taking their medication every day you could see that they've opened up.’ (P. 1; female, age 28, tertiary/higher level education).

The idea of having ‘normal’ lungs without asthma was desired. Providing feedback from adherence in relation to normal lung function may increase adherence behaviour to achieve this sense of normality:

the preventer brings you closer to normal lung functioning.. that's a motivator … So if you've been taking it, and it said your lungs are now X amount of capacity, or functioning the same as if, you didn't have asthma. (P. 5; male, age 28, tertiary/higher level education)

## Discussion

The findings from this study provide insight into young adult preferences for ICS adherence supports and DHI features to provide these supports, in order to improve adherence behaviour within this population. Preferences for adherence supports and DHI features to deliver these included: enabling young adults to take responsibility for adherence and self-management with a prescription refill reminder, supporting habitual adherence with a medication reminder, and providing three levels of information. First, education through images/videos from HCPs with concise text about asthma and ICS. Second, self-monitoring information through an adherence chart, symptom and trigger monitoring, and rewards for adherence. Third, feedback on adherence outcomes by presenting visuals of lungs before and after adherence and lung function monitoring. Those who may not be interested in using DHIs indicated a lack of perceived necessity of ICS and severity of asthma, which have been consistently associated with adherence in this condition (Lycett et al., 2018). This indicates that DHIs are not necessarily suitable to all young adults for whom alternative interventions may be required such as a patient-HCP discussion.

The identified preferences are consistent with factors which influence the uptake of and engagement with general health and well-being apps among adults (Szinay, Jones, Chadborn, Brown, & Naughton, 2020) and with those of young adults in the context of chronic disease (Bendixen, Fairman, Karavolis, Sullivan, & Parmanto, 2017; Saberi, Siedle-Khan, Sheon, & Lightfoot, 2016). However, there appears to be some differences with DHI preferences of chronically ill adults. Namely, incentivisation, such as providing rewards through gamification have been negatively evaluated by adults with arthritis, who value intrinsic as opposed to extrinsic motivation to self-manage their disease as most important, but felt this feature may be useful for younger populations (Geuens, Geurts, Swinnen, Westhovens, & Abeele, 2019; Hilliard, Hahn, Ridge, Eakin, & Riekert, 2014). This difference between disease and age cohorts is likely due to a contrast in perceived disease severity and features that are more developmentally appropriate to younger populations.

A reminder to take and refill ICS was suggested as a feature to overcome forgetfulness, a major barrier to their adherence, and to form habitual ICS-taking behaviour. This is consistent with previous research (Carpenter, Geryk, Sage, Arrindell, & Sleath, 2016; Doyle et al., 2019; Panzera et al., 2013) and has been proposed as an essential feature to include in an app for busy young people (Davis et al., 2019). Habits are formed through repeated performance of the behaviour in response to a specific cue within a stable context (Gardner, 2015). Therefore, it is unsurprising that young adults require the support of an external cue to develop and trigger habitual adherence given their typically unstable routines involving multiple diverse and precarious living contexts, e.g. less than 7% of those under age 25 own their own homes in Ireland and short-term tenancy is the norm for most young people living outside of the family home (Central Statistics Office (CSO), 2016). These prevailing social conditions are unlikely to be conducive to the establishment of good health self-management routines early in adulthood. Smartphone app reminders may serve as external cues to support habitual adherence behaviour, particularly in the early stage of development (Stawarz, Cox, & Blandford, 2015). These interventions have recently been proposed as ideal solutions to provide medication reminders and support the development of habitual adherence behaviour in chronic disease populations (Badawy, Shah, Beg, & Heneghan, 2020). Moreover, stronger habits have been associated with increased adherence in asthma (Bolman, Arwert, & Völlink, 2011). Additionally, delivering a prescription refill reminder in advance may prevent running out of ICS and subsequent non-adherence. Therefore, a DHI that supports habit formation and medication renewal via reminders has significant potential to increase adherence in young adults with asthma.

Young adults want access to general and personal asthma information in a DHI. Interventions combining these levels of information have demonstrated increased adherence and asthma control compared to education (Burgess, Sly, & Devadason, 2010; Gibson et al., 2002) or self-monitoring alone (Janson, McGrath, Covington, Cheng, & Boushey, 2009), and so may provide the most effective information to support adherence. HCPs have indicated substantial support for DHIs for asthma self-management in adolescents (Kosse, Bouvy, de Vries, & Koster, 2019) and adults (Mosnaim et al., 2021; Simpson et al., 2017). Consistent with the current findings, HCPs advocate features such as medication adherence, symptom and asthma control monitoring, with the addition of alerts for deteriorating asthma control and when to seek medical attention (Simpson et al., 2017). However, it would be interesting to explore their perspectives on DHIs for young adults with asthma specifically, particularly in relation to their preferred levels of information, for example, would they provide any additional information if they were in clinic with young adults. HCP perspectives on DHIs are becoming increasingly important with the introduction of Digital Healthcare Legislation in certain European countries (Federal Institute for Drugs and Medical Devices, 2019; mHealthBELGIUM, 2021), under which HCPs can prescribe DHIs which qualify for patient reimbursement.

Lack of knowledge of the ICS adherence-outcome relationship was reported as a barrier to adherence. A recent theoretical review linking medication adherence to causal learning offers an explanation of this barrier. It proposes that patients form beliefs about the medication including its’ necessity, side effects and effectiveness, based on their experience with taking it (Rottman, Marcum, Thorpe, & Gellad, 2017). It is difficult to acquire this cause–effect relation when the medication is not immediately effective and the disease can be asymptomatic (Rottman, 2016), as with ICS and asthma. This may cause non-adherence. Non-adherence disrupts learning about the medication as beliefs, adherence and experience engage in a cyclical process, which can further decrease adherence (Rottman et al., 2017). That is, believing that the medication is not effective would lead to decreased adherence and subsequent decreased effectiveness of the medication, thus reinforcing the negative belief. Having access to personal feedback on the effectiveness of ICS on asthma outcomes within a DHI, would enable patients to learn this cause–effect relation which they may not identify from the medication alone.

### Strengths and limitations

Certain limitations warrant consideration. Difficulty with recruitment limited sample variation in gender and education, and although a range of asthma control and adherence to ICS were reported, most young adults indicated having controlled asthma and non-adherent behaviour. Additionally, as participants self-selected to partake in this research the sample may include those most interested in adherence and/or using DHIs. Future research may benefit from recruiting a larger sample of young adults with increased variation in these important domains.

Notwithstanding these limitations, this appears to be the first study to explore young adult preferences for DHIs to support ICS adherence beyond a specific ethnicity, level of adherence and asthma control (Jeminiwa et al., 2019; Miller et al., 2017; Unni et al., 2018). The sample encapsulated the complete young adult age range and a range of medication adherence which are likely important in shaping young adults’ experience of living with this condition. Finally, this research is now particularly relevant given the recent updates to GINA guidelines. The current GINA guidelines recommend the use of ICS-containing medication as both maintenance and reliever therapy (MART) in patients with moderate to severe asthma, and as reliever therapy in patients with mild asthma (GINA, 2021). This represents a fundamental change from the previously recommended Short-Acting Beta-Agonist as reliever therapy for all patients, due to improved asthma outcomes from using ICS for this purpose (GINA, 2019; Kaplan et al., 2020). These recent updates increase the critical importance of ICS therapy in both controlling and/or managing symptoms in all of those living with the condition. As a result, ICS adherence supports including information about the medication may be needed by an increasing proportion of young adults living with asthma.

## Conclusion

This study provides new insights for understanding appropriate adherence supports at this understudied and critically important stage of the lifespan when lifelong asthma self-care routines become established. Overall, the findings indicate that ICS adherence is challenging and that a DHI may potentially support this behaviour, particularly if development involves young adults living with asthma. These findings enrich our knowledge of young adult preferences for supports that should be included in these interventions and how these can be delivered by DHI features. Ultimately DHIs may have untapped potential to play an important role in optimising the medical treatment of asthma for some patients. Future programmes of work should focus on systematically evaluating the efficacy of these interventions to reduce morbidity and to enhance quality of life for young adults living with asthma.

## Appendices

### Appendix 1: COREQ (COnsolidated criteria for REporting Qualitative research) Checklist

**Table:**

Topic	Item No.	Guide Questions/Description	Reported on Page No.
Domain 1: Research team and reflexivity			
*Personal characteristics*			
Interviewer/facilitator	1	Which author/s conducted the interview or focus group?	4
Credentials	2	What were the researcher’s credentials? E.g. PhD, MD	4
Occupation	3	What was their occupation at the time of the study?	4
Gender	4	Was the researcher male or female?	4
Experience and training	5	What experience or training did the researcher have?	4
*Relationship with participants*			
Relationship established	6	Was a relationship established prior to study commencement?	4
Participant knowledge of the interviewer	7	What did the participants know about the researcher? e.g. personal goals, reasons for doing the research	4
Interviewer characteristics	8	What characteristics were reported about the inter viewer/facilitator? e.g. Bias, assumptions, reasons and interests in the research topic	4
Domain 2: Study design			
*Theoretical framework*			
Methodological orientation and Theory	9	What methodological orientation was stated to underpin the study? e.g. grounded theory, discourse analysis, ethnography, phenomenology, content analysis	4
*Participant selection*			
Sampling	10	How were participants selected? e.g. purposive, convenience, consecutive, snowball	3
Method of approach	11	How were participants approached? e.g. face-to-face, telephone, mail, email	3
Sample size	12	How many participants were in the study?	4
Non-participation	13	How many people refused to participate or dropped out? Reasons?	4
*Setting*			
Setting of data collection	14	Where was the data collected? e.g. home, clinic, workplace	4
Presence of nonparticipants	15	Was anyone else present besides the participants and researchers?	4
Description of sample	16	What are the important characteristics of the sample? e.g. demographic data, date	Table 1, Pg. 17
*Data collection*			
Interview guide	17	Were questions, prompts, guides provided by the authors? Was it pilot tested?	Appendix 2
Repeat interviews	18	Were repeat inter views carried out? If yes, how many?	N/A
Audio/visual recording	19	Did the research use audio or visual recording to collect the data?	4
Field notes	20	Were field notes made during and/or after the inter view or focus group?	4
Duration	21	What was the duration of the inter views or focus group?	4–5
Data saturation	22	Was data saturation discussed?	3
Transcripts returned	23	Were transcripts returned to participants for comment and/or correction?	4
Domain 3: analysis and findings			
*Data analysis*			
Number of data coders	24	How many data coders coded the data?	4
Description of the coding tree	25	Did authors provide a description of the coding tree?	4
Derivation of themes	26	Were themes identified in advance or derived from the data?	4
Software	27	What software, if applicable, was used to manage the data?	4
Participant checking	28	Did participants provide feedback on the findings?	4
*Reporting*			
Quotations presented	29	Were participant quotations presented to illustrate the themes/findings? Was each quotation identified? e.g. participant number	4–8
Data and findings consistent	30	Was there consistency between the data presented and the findings?	4–8
Clarity of major themes	31	Were major themes clearly presented in the findings?	4–8
Clarity of minor themes	32	Is there a description of diverse cases or discussion of minor themes?	4–8

Developed from: Tong A et al. (2007).

### Appendix 2: interview guide

I am hoping to do two things in this interview. Firstly, to hear from you about how you manage your asthma and how you use your preventer inhaler. And secondly, to discuss the potential for digital technology like a smartphone reminder, app, or website to help with your asthma management and taking your preventer.

- First of all, how do you find taking your preventer inhaler?

***Now I am going to ask you some questions about your experience of using technology to support your health.***

o Tell me about any kind of technology you use to help you manage your health? E.g. Fitbit, app, website, reminder.
*If not:*
o Why do you think you don’t use technology for this?
*If yes:*
o Tell me about what you like/dislike about using these technologies? E.g. any specific features?
o Have you ever used any technology to help you manage your asthma?
*If not:*
o Why do you think you don’t use technology for this?
*If yes:*
o If they help, how do these technologies help?
o Would you find managing your asthma difficult without it?
o Would you be interested in using technology to help you take your preventer inhaler?
o What kind of technology would you be more likely to use for this? E.g. smartphone app, website, reminder, Bluetooth sensor attached to your inhaler

***Certain factors may influence whether or not a person takes their preventer inhaler as prescribed. I am hoping to discuss these with you.***

1. ***One of these factors is not having a routine for taking the medication.***
  ➢ Tell me about your routine for taking your preventer
  *If participant seems to have a routine, follow up with:*
  ➢ How did you get into or start this routine? What helped you?
  *If participant does not seem to have a routine for this, ask about other medication routines*
  ➢ Tell me about any routine you have, or had previously, for taking other medication
  *Or*
  ➢ Tell me about other regular routines you have, like something you do everyday?
  ➢ What helped you get into this routine?
  ➢ What would be the best way to help people build a routine or habit, for taking their inhaler, in a digital technology?
2. ***The second factor is a person’s knowledge about asthma medication.***
  ➢ Tell me about any information about asthma or asthma medication, you may be interested in
  ➢ What would be the best way to present this, in a digital technology?
3. ***Some people may believe there is no need to take your preventer inhaler every day.***
  ➢ What do you think about this?
  ➢ Would you like more information about why it is important to take your inhaler every day?
  ➢ If so, what would be the best way to provide this in a digital technology?
4. ***Some people may have concerns about preventer inhalers, like possible side effects.***
  ➢ How do you feel about this?
  ➢ Tell me about any information about potential concerns or side effects, you may be interested in
  ➢ What would be the best way to present this information in a digital technology?
5. ***Some people may not be confident that they’re using or taking their inhaler correctly, for example, they may not be sure if they’re inhaling the medication the right way***
  ➢ How do you feel about this?
  ➢ Tell me about any information on using your inhaler, that you may be interested in?
  ➢ What would be the best way to present this, in a digital technology?
  ❖ Are there any other barriers you have, or had previously, to taking your preventer as prescribed?
  ❖ Is there anything else that helps you take your preventer?
  ❖ Is there anything else you would like to discuss? Or any other comments or suggestions you would like to make?

### Appendix 3: demographics and asthma characteristics collected

**Table:**

**Demographics**
What is your age (in years)?
Please specify your gender:
❑ Female
❑ Male
❑ Other
❑ Prefer not to say
Please indicate your ethnic background:
❑ White: Irish
❑ White: Irish Traveller
❑ Any other white background
❑ Black/Black Irish
❑ Asian/Asian Irish
❑ Mixed/Other
What is the highest level of education you have completed?
❑ Primary level
❑ Secondary level
❑ Tertiary/Higher level
Do you currently have a medical card?
❑ Yes
❑ No
Have you visited the Emergency Department because of an asthma exacerbation in the past year?
❑ Yes
❑ No
Have you been admitted to hospital because of an asthma exacerbation in the past year?
❑ Yes
❑ No
Have you been treated with oral steroids for your asthma in the past year?
❑ Yes
❑ No
**Asthma Control Test**
1. During the last 4 weeks, how much of the time has your asthma kept you from getting as much done at work, school or home?
❑ All of the time
❑ Most of the time
❑ Some of the time
❑ A little of the time
❑ None of the time
2. During the last 4 weeks, how often have you had shortness of breath?
❑ More than once a day
❑ Once a day
❑ 3–6 times a week
❑ Once or twice a week
❑ Not at all
3. During the last 4 weeks, how often have your asthma symptoms (wheezing, coughing, shortness of breath, chest tightness or pain) woken you up at night or earlier than usual in the morning?
❑ 4 or more nights a week
❑ 2 or 3 nights a week
❑ Once a week
❑ Once or twice
❑ Not at all
4. During the last 4 weeks, how often have you used your rescue inhaler or nebuliser medication (such as Salbutamol)?
❑ 3 or more times per day
❑ Once or twice per day
❑ 2 or 3 times per week
❑ Once a week or less
❑ Not at all
5. How would you rate your asthma control during the last 4 weeks?
❑ Not controlled at all
❑ Poorly controlled
❑ Somewhat controlled
❑ Well controlled
**5-item Medication Adherence Report Scale (MARS-5)**
1. I forget to take my preventer inhaler
❑ Never
❑ Rarely
❑ Sometimes
❑ Often
❑ Always
2. I alter the dose of my preventer inhaler
❑ Never
❑ Rarely
❑ Sometimes
❑ Often
❑ Always
3. I stop taking my preventer for a while when I am not supposed to
❑ Never
❑ Rarely
❑ Sometimes
❑ Often
❑ Always
4. I decide to miss out a dose of my preventer inhaler
❑ Never
❑ Rarely
❑ Sometimes
❑ Often
❑ Always
5. I take less (of my preventer) than instructed
❑ Never
❑ Rarely
❑ Sometimes
❑ Often
❑ Always
